# Biologic disease modifying antirheumatic drugs and Janus kinase inhibitors in paediatric rheumatology – what we know and what we do not know from randomized controlled trials

**DOI:** 10.1186/s12969-021-00514-4

**Published:** 2021-03-25

**Authors:** Tatjana Welzel, Carolyn Winskill, Nancy Zhang, Andreas Woerner, Marc Pfister

**Affiliations:** 1 Paediatric Pharmacology and Pharmacometrics, University Children’s Hospital Basel (UKBB), University of Basel, Spitalstrasse 33, CH 4031 Basel, Switzerland; 2 Paediatric Rheumatology, University Children’s Hospital Basel (UKBB), University of Basel, Basel, Switzerland; 3 Integrated Drug Development, Certara LP, Princeton, NJ USA

**Keywords:** Randomized controlled trials, Paediatric rheumatology, Monoclonal antibodies, Efficacy, Safety, Pharmacokinetics

## Abstract

**Background:**

Biologic disease modifying antirheumatic drugs (bDMARDs) and Janus Kinase (JAK) inhibitors are prescribed in adult and paediatric rheumatology. Due to age-dependent changes, disease course, and pharmacokinetic processes paediatric patients with inflammatory rheumatic diseases (PiRD) differ from adult rheumatology patients.

**Methods:**

A systematic literature search for randomized clinical trials (RCTs) in PiRD treated with bDMARDs/JAK inhibitors was conducted on Medline, clinicaltrials.gov, clinicaltrialsregister.eu and conference abstracts as of July 2020. RCTs were included if (i) patients were aged ≤20 years, (ii) patients had a predefined rheumatic diagnosis and (iii) RCT reported predefined outcomes. Selected studies were excluded in case of (i) observational or single arm study or (ii) sample size ≤5 patients. Study characteristics were extracted.

**Results:**

Out of 608 screened references, 65 references were selected, reporting 35 unique RCTs. All 35 RCTs reported efficacy while 34/35 provided safety outcomes and 16/35 provided pharmacokinetic data. The most common investigated treatments were TNF inhibitors (60%), IL-1 inhibitors (17%) and IL-6 inhibitors (9%). No RCTs with published results were identified for baricitinib, brodalumab, certolizumab pegol, guselkumab, risankizumab, rituximab, sarilumab, secukinumab, tildrakizumab, or upadacitinib. In patients with juvenile idiopathic arthritis (JIA) 25/35 RCTs were conducted. The remaining 10 RCTs were performed in non-JIA patients including plaque psoriasis, Kawasaki Disease, systemic lupus erythematosus and non-infectious uveitis. In JIA-RCTs, the control arm was mainly placebo and the concomitant treatments were either methotrexate, non-steroidal anti-inflammatory drugs (NSAID) or corticosteroids. Non-JIA patients mostly received NSAID. There are ongoing trials investigating abatacept, adalimumab, baricitinib, brodalumab, certolizumab pegol, etanercept, guselkumab, infliximab, risankizumab, secukinumab, tofacitinib and tildrakizumab.

**Conclusion:**

Despite the FDA Modernization Act and support of major paediatric rheumatology networks, such as the Pediatric Rheumatology Collaborative Study Group (PRCSG) and the Paediatric Rheumatology International Trials Organization (PRINTO), which resulted in drug approval for PiRD indications, there are limited RCTs in PiRD patients. As therapy response is influenced by age-dependent changes, pharmacokinetic processes and disease course it is important to consider developmental changes in bDMARDs/JAK inhibitor use in PiRD patients. As such it is critical to collaborate and conduct international RCTs to appropriately investigate and characterize efficacy, safety and pharmacokinetics of bDMARDs/JAK inhibitors in paediatric rheumatology.

**Supplementary Information:**

The online version contains supplementary material available at 10.1186/s12969-021-00514-4.

## Background

Paediatric inflammatory rheumatic diseases (PiRDs) are complex rare chronic inflammatory conditions with risk of chronic morbidity and mortality affecting infants, children and adolescents [1]. PiRDs include different heterogeneous disease groups, such as the juvenile idiopathic arthritis (JIA), connective tissue diseases (CTD), systemic lupus erythematosus (SLE), vasculitis, uveitis and autoinflammatory diseases (AID). JIA is one of the most common PiRD groups and can be divided into different subgroups according to the International League of Associations for Rheumatology (ILAR) [2, 3]. In paediatric rheumatology, (i) responsive and valid instruments to assess disease activity and (ii) standardized outcome measurements are important to achieve defined treatment aims, to avoid disease burden and to optimize patients care [4–7]. Treatment aims in PiRD patients include control of systemic inflammation, prevention of structural damage, avoidance of disease comorbidities and drug toxicities, improvement of physiological growth and development, increase of the quality of life and enabling participation in social life. To achieve these treatment goals, treat-to-target (T2T) strategies similar to those used in adult rheumatology have been implemented in PiRD management [8–10]. To reach the defined treatment targets, different levels of disease activity require different treatment selections and dose adjustments [11]. The cytokine modulating effects of biologic disease modifying antirheumatic drugs (bDMARDs) or Janus kinase (JAK) inhibitors have enabled T2T strategies, since they allow important inflammatory disease pathways to be targeted [12–15]. Over the past 15 years, bDMARDs use has become essential in paediatric rheumatology and has markedly improved clinical outcomes [13, 15–19]. However, off-label use in PiRD patients is still common [20–26].

Although some rheumatologic diseases occur in paediatric and adult patients, considerable differences in disease symptoms, disease course and disease activity might exist [27–32]. Moreover, some PiRDs are not common or well known in adulthood [33–36]. For example, the JIA associated uveitis is the most frequent and potentially the most devastating extra-articular manifestation of the JIA, commonly affecting children aged 3 to 7 years [37]. Additionally, Kawasaki Disease (KD) is an acute inflammatory febrile vasculitis of mainly medium-sized arteries that typically affects children younger than 5 years [38]. Furthermore, PiRD patients differ from adult rheumatology patients in several physiological aspects, due to age-dependent changes, maturation processes, differences in body composition and pharmacokinetic (PK) processes, such as drug absorption, distribution, metabolism, and excretion [39–45]. All these aspects are important factors to consider in diagnosis and treatment. This highlights that paediatric drug development cannot simply mimic development strategies for adults, but has to respect paediatric pathophysiology and specific paediatric disease characteristics [46]. Nevertheless, it is common to use the same bDMARDs and JAK inhibitors in paediatric and adult rheumatology, and most paediatric trials and dosing regimens are performed on the basis of existing adult data [47].

The goal of this review is to assess the current state of knowledge obtained from previously performed randomized controlled trials (RCTs) in PiRD patients treated with bDMARDs and JAK inhibitors. In addition, an overview of approved bDMARDs and JAK inhibitors from the Food and Drug Administration (FDA) and European Medicines Agency (EMA) in paediatric and adult rheumatology is provided.

## Methods

### Information sources and search

A systemic literature search was conducted on Medline via PubMed, the US National Institutes of Health Ongoing Trials Register ClinicalTrials.gov (www.clinicaltrials.gov), and the EU Clinical Trials Register (www.clinicaltrialsregister.eu). The MeSH terms used for electronic search on PubMed and ClinicalTrials.gov are detailed in the supplementary material (supplementary data S1). Similar search terms were used for the EU Clinical Trials Register. The statistical and clinical sections of the New Drug Approval (NDA) web pages of regulatory authorities in the US and Europe were reviewed for approved drugs (www.fda.gov, www.ema.europa.eu). Abstract searches were conducted after conferences, including the American College of Rheumatology (ACR), the European League Against Rheumatism (EULAR), the International Society of Systemic Auto-Inflammatory Diseases (ISSAID), Société Francophone pour la Rhumatologie et les Maladies Inflammatoires en Pédiatrie (SOFREMIP) and the Pediatric Rheumatology European Society (PRES). Additionally, relevant studies were identified by manual search of the bibliographies of references retrieved from PubMed. For all literature sources, only English articles were screened.

### Eligiblity and exclusion criteria

RCTs of patients aged 20 years and younger treated with predefined bDMARDs/JAK inhibitors were included if the sample size was at least five patients, and if PiRD diagnosis had been confirmed. The PiRDs included are listed in Table 1. The drugs and drug classes reviewed included the following: (i) Anti-CD20 agents: rituximab; (ii) CD80/86 inhibitors: abatacept; (iii) IL-1 inhibitors: anakinra, canakinumab, rilonacept; (iv) IL-6 inhibitors: tocilizumab, sarilumab; (v) IL-12/23 inhibitors: ustekinumab; (vi) IL-23 inhibitors: guselkumab, risankizumab, tildrakizumab; (vii) IL-17 inhibitors: secukinumab, ixekizumab, brodalumab; (viii) Tumour necrosis factor (TNF) inhibitors: adalimumab, etanercept, golimumab, infliximab, certolizumab pegol; (ix) BAFF inhibitors: belimumab; (x) JAK inhibitors: baricitinib, tofacitinib, upadacitinib. Studies must have at least included a relevant primary or secondary efficacy endpoint/outcome as detailed in the supplementary material (supplementary data S3). Consequently, studies were excluded if (i) the indication was not relevant, (ii) the population was not relevant, including studies which enrolled both adults and children with PiRD, (iii) the study design was not relevant including observational studies, single arm studies, reviews, meta-analyses and pooled analysis of multiple RCTs, (iv) treatment was not that as predefined, (v) the endpoint/outcome was not relevant, and/or (vi) the report was a duplicate of prior published results without any additional information.

**Table 1:** Defined diagnoses of paediatric inflammatory rheumatic diseases (PiRD) for literature search

Indication	Population
**JIA**	Polyarticular rheumatoid factor positive/negative JIA (PJIA)
	Persistent or extended oligoarticular JIA (OJIA)
	Enthesitis-related juvenile idiopathic arthritis and juvenile ankylosing spondylitis, including sacroiliitis (ERA)
	Psoriatic juvenile idiopathic arthritis (PsA)
	Systemic JIA (SJIA)
**Uveitis**	JIA-associated uveitis
	Non-infectious uveitis
**Autoinflammatory Diseases**	Familial Mediterranean Fever (FMF)
	TNF receptor-1 associated periodic syndrome (TRAPS)
	Cryopyrin-associated periodic syndromes (CAPS)
	Mevalonate Kinase Deficiency (MKD)/Hyperimmunoglobulin D syndrome (HIDS)
	Unclassified periodic fever syndromes
	Chronic recurrent multifocal osteomyelitis (CRMO) and Majeed syndrome
	Deficiency of the interleukin-1 receptor antagonist (DIRA)
	A20 haploinsufficiency (HA20)
	Sideroblastic anemia with B cell immunodeficiency, periodic fevers and developmental delay syndrome (SIFD)
	Pyogenic arthritis, pyoderma gangraenosum and acne (PAPA)
	Deficiency of the interleukin-36 receptor antagonist (DITRA)
	Palmar plantar pustulosis (PPP)
	Pyoderma gangraenosum
**Interferonopathy**	Chronic atypical neutrophilic dermatitis with lipodystrophy and elevated temperature (CANDLE) Stimulator of interferon genes-associated vasculopathy with onset in infancy (SAVI)
**Vasculitis**	Takayasu arteritis
	Leucocytoclastic vasculitis
	Granulomatosis with polyangiitis (GPA), Wegener’s Granulomatosis
	Polyarteritis nodosa
	Microscopic polyangiitis (MPA)
	Eosinophilic granulomatosis with polyangiitis
	Kawasaki Disease (KD)
	Behcet disease
**Connective Tissue Diseases**	Systemic Lupus Erythematosus (SLE)
	Juvenile Dermatomyositis (JDM)
	Paediatric sarcoidosis
	Systemic and Localized Scleroderma
	Sjögren Syndrome
	Mixed connective tissue diseases (MCTD)
**Macrophage activation syndrome**	
**Psoriasis**	

### Study selection and data collection process

Initial screening, based on retrieved abstracts, as well as the eligibility assessment based on full-text publications were performed by two independent review authors according to a review protocol (supplementary data S2). One scientist was responsible for the execution and documentation, and the other provided support as therapeutic area expert. Any discrepancies were resolved through discussion or consultation with a third independent reviewer. The primary reason for exclusion was documented for each excluded reference. Aggregate (summary) level data were extracted from each included trial by two independent review authors. The defined extracted variables for each RCTs included baseline demographic and clinical characteristics such as study design, location, patient population, sample size, age criteria, treatment and primary outcome/endpoint.

## Results

### Study selection

**Fig. 1:**
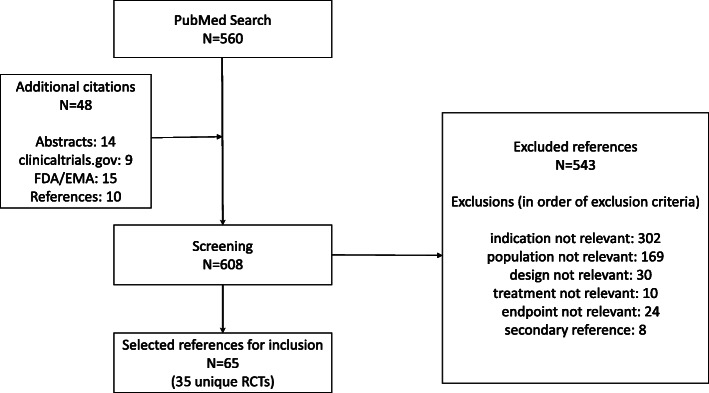
Flow chart of study selection and reasons for study exclusion after literature search

A systematic literature search was performed on July 26, 2020 using the predefined search criteria. A total of 608 references were screened, and 65 references for 35 uniquely identified RCTs performed in PiRD patients were selected for inclusion (Fig. 1). Of the references excluded, the large majority were due to irrelevant indication (56%, 302/543) or population (31%, 169/543). In total, the majority of RCTs were performed for TNF inhibitors (60%), IL-1 inhibitors (17%) and IL-6 inhibitors (9%). Only one RCT was available for the BAFF inhibitor belimumab, the JAK inhibitor tofacitinib, the IL-12/23 inhibitor ustekinumab, the IL-17 inhibitor ixekizumab, the TNF inhibitor golimumab and the CD 80/86 inhibitor abatacept. No RCTs with published results were identified for the anti-CD20 agent rituximab, the TNF inhibitor certolizumab pegol, the IL-6 inhibitor sarilumab, the IL-17 inhibitors brodalumab and secukinumab, the IL-23 inhibitors guselkumab, risankizumab and tildrakizumab and the JAK inhibitors baricitinib and upadacitinib (Table 2). Currently, there are no recruiting RCTs for sarilumab but there are two ongoing single-arm PK studies in sarilumab, NCT02776735 and NCT02991469 (data not shown). For the JAK inhibitors three Phase III RCTs are investigating baricitinib, and one Phase III global RCT is investigating tofacitinib in JIA patients (Table 3). Furthermore, there are ongoing studies for guselkumab, risankizumab, tildrakizumab, brodalumab, secukinumab and certolizumab pegol mainly in PiRD patients with psoriasis (Table 3). Additionally, some studies are recruiting to investigate adalimumab in JIA-associated uveitis, abatacept in OJIA, infliximab in KD and etanercept in OJIA and PJIA.

**Table 2:** Overview of completed randomized controlled trials performed in paediatric rheumatology patients treated with bDMARDs/JAK inhibitors (July 2020)

Drug Class	Drug	Studies	Arms	Patients	Indications	Populations
Anti-CD 20 agent	rituximab	0				
CD80/86 inhibitor	abatacept	1	2	190	JIA	PJIA, SJIA (without systemic features), extended OJIA
IL-1 inhibitor	anakinra	2	4	110	JIA	PJIA, SJIA
	canakinumab	2	4	261	JIA	SJIA
	rilonacept	2	5	95	JIA	SJIA
IL-6 inhibitor	sarilumab	0				
	tocilizumab	3	6	356	JIA	PJIA, extended OJIA, SJIA
IL-12/23 inhibitor	ustekinumab	1	3	110	Psoriasis	Plaque psoriasis
IL-23 inhibitor	guselkumab	0				
	risankizumab	0				
	tildrakizumab	0				
IL-17 inhibitor	brodalumab	0				
	ixekizumab	1	3	201	Psoriasis	Plaque psoriasis
	secukinumab	0				
TNF inhibitor	adalimumab	6	13	485	JIA, psoriasis, uveitis	ERA, JIA-associated uveitis, PJIA, plaque psoriasis
	etanercept	8	17	746	JIA, psoriasis, vasculitis	PJIA, OJIA, PsA, ERA, SJIA, plaque psoriasis, KD
	golimumab	1	2	154	JIA	PJIA, SJIA (without systemic features), PsA
	infliximab	6	13	576	JIA, uveitis, vasculitis	PJIA, non-infectious uveitis, KD
	certolizumab pegol	0				
BAFF inhibitor	belimumab	1	2	93	CTD	SLE
JAK inhibitor	tofacitinib	1	2	225	JIA	ERA, PJIA, PsA
	baricitinib	0				
	upadacitinib	0				

*Abbreviations: IL* interleukin, *TNF* tumour necrosis factor, *JAK* Janus Kinase, *JIA* juvenile idiopathic arthritis, *CTD* connective tissue disease, *PJIA* polyarticular juvenile idiopathic arthritis, *KD* Kawasaki disease, *SJIA* systemic juvenile idiopathic arthritis, *OJIA* oligoarticular juvenile idiopathic arthritis, *ERA* enthesitis-related juvenile idiopathic arthritis, *PsA* psoriatic juvenile idiopathic arthritis, *SLE* systemic lupus erythematosus

**Table 3:** Ongoing or recruiting studies in paediatric patients with inflammatory rheumatic diseases (July 2020)

Drug class	Drug	Study	Sponsor	Population	Region	Study duration	Primary outcome/endpoint
CD 80/86 inhibitor	abatacept	Limit-JIA, NCT03841357	Duke University	OJIA	United States	10/2019–12/2022	Joint count, active anterior uveitis
IL-23 inhibitor	guselkumab	^a^PROTOSTAR, NCT03451851	Janssen	Paediatric psoriasis	global	7/2018–6/2025	PASI75, PGA ≤1
	risankizumab	^b^M19–977, NCT04435600	Abbvie	Paediatric psoriasis	United States	7/2020–06/2025	PASI75; PGA ≤ 1
	tildrakizumab	TILD-19-12, NCT03997786	Sun Pharma Global FZE	Paediatric psoriasis	United States	1/2020–11/2023	PASI75, PGA ≤1
IL-17 inhibitor	brodalumab	^c^EMBRACE 1, NCT04305327	LEO Pharma	Paediatric psoriasis	global	9/2020–11/2023	PASI75
	secukinumab	^d^CAIN457F2304, NCT03031782	Novartis	PsA, ERA	global	5/2017–12/2020	Disease flare
		^e^CAIN457A2311, NCT03668613	Novartis	Paediatric psoriasis	global	8/2018–9/2023	PASI75, PGA ≤1
		^f^CAIN457A2310, NCT02471144	Novartis	Paediatric psoriasis	global	9/2015–7/2023	PASI75, PGA ≤1
TNF inhibitor	adalimumab	^g^ADJUST, NCT03816397	UCSF	JIA-associated uveitis	United States	12/2019–12/2022	Treatment failure
	etanercept	STARS, EudraCT 2018–001931-27	IRCCS Istituto Giannina Gaslini	OJIA, PJIA	Italy	NA	Clinical inactive disease
	infliximab	KIDCARE, NCT03065244	UCSD	Kawasaki disease	United States	2/2017–9/2020	Fever
	certolizumab pegol	CIMcare, NCT04123795	UCB Biopharma	Paediatric psoriasis	North America	1/2020–4/2023	PASI75, PGA ≤1
JAK inhibitor	baricitinib	^h^JUVE-BRIGHT, NCT04088409	Eli Lilly	JIA-associated uveitis	Europe	10/2019–7/2022	Uveitis disease response
		^i^JUVE-BALM, NCT04088396	Eli Lilly	SJIA	global	2/2020–4/2023	Disease flare
		^j^JUVE-BASIS, NCT03773978	Eli Lilly	PJIA, extended OJIA, ERA, PsA	global	12/2018–8/2021	Disease flare
	tofacitinib	A3921165, NCT03000439	Pfizer	SJIA	global	5/2018–8/2023	Disease flare

*Abbreviations*: *IL* interleukin, *TNF* tumour necrosis factor, *JAK* Janus Kinase, *ERA* enthesitis-related juvenile idiopathic arthritis, *JIA* juvenile idiopathic arthritis, *PsA*, psoriatic juvenile idiopathic arthritis, *OJIA* oligoarticular juvenile idiopathic arthritis, *PASI* psoriasis area and severity index, *PGA* Physician global assessment, *PJIA* polyarticular juvenile idiopathic arthritis, *SJIA* systemic juvenile idiopathic arthritis, *NA* not applicable

^a^Also registered under EudraCT 2017–003053-42; ^b^Also registered under EudraCT 2019–004141-32, ^c^Also registered under EudraCT 2019–001868-30; ^d^Also registered under EudraCT 2016–003761-26; ^e^Also registered under EudraCT 2017–004515-39; ^f^Also registered under EudraCT 2014–005663-32; ^g^Also registered under EudraCT 2019–000412-29; ^h^Also registered under EudraCT 2019–00119-10; ^i^Also registered under EudraCT 2017–004495-60; ^j^Also registered under EudraCT 2017–004518-24

### Study characteristics

Approximately two-thirds (25 out of 35) of the identified RCTs were conducted in JIA patients and the remaining ten RCTs were performed in non-JIA patients, including KD, plaque psoriasis, SLE, and non-infectious uveitis (Tables 4 and 5). The mean/median age of children enrolled in the JIA RCTs ranged from 8 years to 15.3 years. In contrast, the non-JIA patients included in RCTs had a mean/median age range varying between 2.2 and 15.2 years, with KD patients being younger (range 2.2 to 3.7 years). In JIA RCTs, the control was mainly placebo, and the concomitant background treatments were usually either methotrexate, NSAID or corticosteroids, whereas in non-JIA trials the control arm was a mixture of placebo or standard of care treatments and patients received mostly NSAID as background treatments (data not shown for the control arm). The primary efficacy outcome/endpoint in the JIA RCTs was mainly ACR Pedi 30/modified ACR Pedi 30 or disease flare (Table 4). Other instruments to assess the primary outcome were count of joints with active arthritis, the assessment of Spondyloarthritis International Society 40% score (ASAS 40), inactive disease, treatment failure and improvement of laser flare photometry (Table 4). In non-JIA patients, efficacy outcomes/endpoints varied due to heterogeneous subgroups. The primary efficacy outcome/endpoint of RCTs in KD was mainly related to fever, whereas for plaque psoriasis the Psoriasis Area and Severity Index (PASI 75), or the Physician Global Assessment (PGA) was used (Table 5). The RCT addressing SLE used the SLR response index (SRI 4), whereas the primary outcome/endpoint in non-infectious uveitis was assessed with uveitis disease activity using the Standardization of Uveitis Nomenclature (SUN) criteria, AC cells and vitreous haze. The majority of the JIA RCTs were global studies or otherwise conducted in either Europe or the United States, with one study (NCT00144599) located in Japan (data not shown). The non-JIA RCTs took place either in North America, Europe or globally (data not shown). In particular, KD RCTs took place mainly in Asia or the United States. Details for JIA and non-JIA RCTs are shown in Tables 4 and 5. All non-JIA studies were of a parallel study design, while in JIA studies there was a mixture of parallel and withdrawal study designs (Tables 4 and 5). The main conclusion of the majority of the studies (in terms of meeting the primary endpoint/outcome) was that the bDMARDs evaluated were more effective in comparison to placebo or standard of care (Tables 4 and 5).

**Table 4:** Overview and general characteristics of identified reviewed randomized controlled trials performed in JIA patients (July 2020)

Drug class	Drug	Dose	Study (phase)	Study time^**c**^	Population	N	Age criteria	Age^**a**^	Background	Primary outcome/endpoint	Main conclusion
**CD80/86 inhibitor**	abatacept	10 mg/kg q4w	^d^IM101–033, NCT00095173 (III) [48–51]^b^	26	PJIA, SJIA (without systemic features), extended OJIA	190	6 to 17	12.4	MTX, corticosteroids	Disease flare	Effective
**IL-1 inhibitor**	anakinra	1 mg/kg/d	^e,b^990758–990779, NCT00037648 (II) [52, 53]	16	PJIA	86	2 to 17	12	MTX	Safety	Efficacy is inconclusive
		2 mg/kg/d	ANAJIS, NCT00339157 (II/III) [53, 54]	4.33	SJIA	24	2 to 20	8.5	NSAID, corticosteroids	Modified ACR Pedi 30	Effective
	canakinumab	4 mg/kg single dose	^f^β-SPECIFIC 1, NCT00886769 (III) [55–57]	4.33	SJIA	84	2 to 19	8^a^	MTX, NSAID, corticosteroids	ACR Pedi 30	Effective
		4 mg/kg q4w	^g^β-SPECIFIC 2, NCT00889863 (III) [55–57]^b^	120	SJIA	177	2 to 19	8^a^	MTX, NSAID, corticosteroids	Disease flare	Effective
	rilonacept	2.2 mg/kg qw	RAPPORT, NCT00534495 (II) [58, 59]	24	SJIA	71	1 to 19	10	NA	Modified ACR Pedi 30	Effective
		2.2 mg/kg qw, 4.4 mg/kg qw	IL1T-AI-0504, NCT01803321 (II) [60]	104	SJIA	24	4 to 20	12.6	MTX, NSAID, corticosteroids	ACR Pedi 30	Not effective
**IL-6 inhibitor**	tocilizumab	8 mg/kg q4w, 10 mg/kg q4w	^h^CHERISH, NCT00988221 (III) [61–65]^b^	104	PJIA, extended OJIA	188	2 to 17	11	MTX, corticosteroids	Disease flare	Effective
		8 mg/kg q2w, 12 mg/kg q2w	^i^TENDER, NCT00642460 (III) [63–67]	260	SJIA	112	2 to 17	9.7	MTX, corticosteroids	Modified ACR Pedi 30	Effective
		8 mg/kg q2w	MRA316JP, NCT00144599 (III) [68]^b^	18	SJIA	56	2 to 19	8.3	corticosteroids	ACR Pedi 30	Effective
**TNF inhibitor**	adalimumab	24 mg/m2 q2w	^j^M11–328, NCT01166282 (III) [69–71]	12	ERA	46	6 to 17	12.9	MTX or SSZ, NSAID	Joints with active arthritis	Effective
		40 mg q2w	Horneff 2012, EudraCT 2007–003358-27(III) [72]	12	ERA	32	12 to 17	15.3	NSAID, corticosteroids	ASAS40	Effective
		24 mg/m2 q2w	^k,b^DE038, NCT00048542 (III) [73, 74]	48	PJIA	171	4 to 17	11.2	MTX, NSAID, corticosteroids	Disease flare	Effective
		20 mg q2w, 40 mg q2w	SYCAMORE, EudraCT 2010–021141-41 (NA) [75, 76]	78	JIA-associated uveitis	90	2 to 18	8.9	MTX	Treatment failure	Effective
		24 mg/m2 q2w, 40 mg q2w	^l^ADJUVITE, NCT01385826 (II/III) [77]	52	JIA-associated uveitis	32	> = 4	9.5^a^	MTX, corticosteroids (oral and topical)	LFP improvement > = 30% and no worsening on slit lamp	Effective
	etanercept	0.8 mg/kg/qw	Horneff 2015, EudraCT 2010–020423-51 (III) [78] ^b^	48	ERA	38	6 to 17	13.3	SSZ, NSAID, corticosteroids	Disease flare	Effective
		0.8 mg/kg/qw	^o^16.0016 (NA) [79, 80]	30.33	PJIA	69	4 to 17	NA	NSAID, corticosteroids	Disease flare	Effective
		0.8 mg/kg/qw	^o^20021616, NCT03780959 (II/III) [81]^b^	30.33	PJIA	69	4 to 18	10.5	NSAID	Disease flare	Effective
		0.8 mg/kg/qw	20021628, NCT03781375 (III) [82]	52	PJIA	25	NA	10.1	MTX	ACR Pedi 30	NA
		0.8 mg/kg/qw	TREAT, NCT00443430 (IV) [83, 84]	52	PJIA	85	2 to 17	10.5	MTX	Clinical inactive disease	Not effective
		0.8 mg/kg/qw, 1.6 mg/kg/qw	^b^20021631, NCT00078806 (III) [85]	39	SJIA	19	2 to 18	9.1	MTX, NSAID, corticosteroids	Disease flare	NA
		0.8 mg/kg/qw	BeSt for Kids, NTR1574 (NA) [86, 87]	12	PJIA,RF^−^; OJIA, JPsA	94	2 to 16	8.8^a^	MTX	Unclear	Effective
	golimumab	30 mg/m2 q4w	^m^GO KIDS, NCT01230827 (III) [88, 89]^b^	48	PJIA, SJIA (without systemic features), PsA	154	2 to 17	11.1	MTX, corticosteroids	Disease flare	Not effective
	infliximab	3 mg/kg, 6 mg/kg	CR004774, NCT00036374 (III) [90]	58	PJIA	122	4 to 17	11.2	MTX, NSAID, corticosteroids	ACR Pedi 30	Not effective
		3–5 mg/kg	ACUTE-JIA, NCT01015547 (III) [91]	54	PJIA	60	4 to 15	9.6	MTX, other DMARDs	ACR Pedi 75	Effective
**JAK inhibitor**	tofacitinib	2–5 mg BID	^n^A3921104, NCT02592434 (III) [92]^b^	44	ERA, PJIA, PsA	225	2 to 17	13^a^	NA	Disease flare	Effective

*Abbreviations:* Drug class: *IL* interleukin, *TNF* tumour necrosis factor, *JAK* Janus Kinase; Dose: *mg* milligram, *kg* kilogram, *d* per day, *qw* once per week, *q2w* once per every 2 weeks, *q4w* once per every 4 weeks, *BID* twice a day; Population: *ERA* enthesitis-related juvenile idiopathic arthritis, *PsA* psoriatic juvenile idiopathic arthritis, *OJIA* oligoarticular juvenile idiopathic arthritis, *PJIA* juvenile Polyarticular idiopathic arthritis, *RF*^−^ rheumatoid factor negative, *SJIA* systemic juvenile idiopathic arthritis; Background: *MTX* methotrexate, *HCQ* hydroxycloroquine, *NSAID* non-steroidal anti-inflammatory drugs, *DMARDS* disease modifying antirheumatic drugs, *SSZ* sulfasalazine; Outcome: *ACR Pedi 30* ACR Pedi 30% response criteria, *ACR Pedi 75* ACR Pedi 75% response criteria, *LFP* laser flare photometry, *ASAS40* assessment in ankylosing spondylitis response criteria 40%; *NA* not available

^a^median age, otherwise mean age across all arms of the study, ^b^withdrawal study design instead of parallel, ^c^duration in weeks, ^d^Also registered under EudraCT 2005–000443-28; ^e^Also registered under EudraCT 2015–002466-22; ^f^Also registered under EudraCT 2008–005476-27; ^g^Also registered under EudraCT 2008–005479-82; ^h^Also registered under EudraCT 2009–011593-15; ^i^Also registered under EudraCT 2007–00872-18; ^j^Also registered under EudraCT 2009–017938-46; ^k^Also registered under EudraCT 2011–001661-40; ^l^Also registered under EudraCT 2010–019441-26; ^m^Also registered under EudraCT 2009–015019-42; ^n^Also registered under EudraCT 2015–001438-46; ^o^same study

**Table 5:** Overview and general characteristics of identified reviewed randomized controlled trials performed in non-JIA patients (July 2020)

Drug class	Drug	Dose	Study (phase)	Studytime^c^	Population	N	Age criteria	Age^a^	Primary outcome/endpoint	Main conclusion
**IL-12/23 inhibitor**	ustekinumab	0.75 mg/kg, 22.5/45/ 90 mg	^d^CADMUS, NCT01090427 (III) [93]^b^	60	Plaque psoriasis	110	12 to 17	15.2	PGA ≤1	Effective
**IL-17 inhibitor**	ixekizumab	20 mg BW < 25 kg q4w, 40 mg BW 25–50 kg q4w, 80 mg BW > 50 kg q4w	^e^IXORA-PEDS, NCT03073200 (III) [94] ^b^	12	Plaque psoriasis	201	6 to 17	13.5	PASI75, PGA ≤1	Effective
**TNF inhibitor**	adalimumab	0.4 mg/kg q2w, 0.8 mg/kg q2w	^f^M04–717, NCT01251614 [95–98] ^b^	52	Plaque psoriasis	114	4 to 17	13	PASI75, PGA ≤1	Effective
	etanercept	0.8 mg/kg/qw	EATAK, NCT00841789 (II) [99] ^b^	6	KD	205	0 to 18	3.7	Fever	Not effective
		0.8 mg/kg/qw	20030211, NCT00078819 (III) [100–104] ^b^	48	Plaque psoriasis	211	4 to 17	13^a^	PASI75	Effective
	infliximab	5 mg/kg single dose	Han 2018 (NA) [105] ^b^	0.571	KD	154	0 to 4	2.2^a^	Unclear	Effective
		5 mg/kg single dose	TA-650-22, NCT01596335 (III) [106] ^b^	8	KD	31	1 to 10	3^a^	Defervescence	Effective
		5 mg/kg single dose	Tremoulet 2014, NCT00760435 (III) [107, 108] ^b^	5	KD	196	0 to 17	3^a^	Fever	Effective
		5 mg/kg, 10 mg/kg q4w	Pro00000057, NCT00589628 (IV) [109] ^b^	39	Non-infectious uveitis	13	4 to 18	NA	Uveitis disease activity	NA
**BAFF inhibitor**	belimumab	10 mg/kg qm	^g^PLUTO, NCT01649765 (II) [110, 111] ^b^	52	SLE	93	5 to 17	14	SRI4	Effective

*Abbreviations:* Drug class: *IL* interleukin, *TNF* tumour necrosis factor; Dose: *mg* milligram, *kg* kilogram, *qw* once per week, *q2w* once per every 2 weeks, *q4w* once per every 4 weeks, *qm* once every month; Population: *KD* Kawasaki disease, *SLE* systemic lupus erythematosus; Outcome: *PASI* psoriasis area and severity index, *PGA* Physician global assessment, *SRI4* systemic lupus erythematosus response index 4, *NA* not available

^a^median age, otherwise mean age across all arms of the study, ^b^parallel study design, ^c^duration in weeks; ^d^Also registered under EudraCT 2009–014368-20, ^e^Also registered under EudraCT 2016–003331-38;^f^Also registered under EudraCT 2009–013072-52; ^g^Also registered under EudraCT 2011–000368-88

### Approved bDMARDs and JAK inhibitors in paediatric and adult rheumatology

In March 2020, the FDA has approved all 23 reviewed drugs, including bDMARDs and JAK inhibitors for adult rheumatology, whereas the EMA has approved 22 (Table 6). For PiRD patients, 10 bDMARDs (EMA) and 11 (FDA) have been approved (Table 6). Not surprisingly, the more recently approved bDMARDs in adult rheumatology and the JAK inhibitors have mostly not yet been approved for PiRD patients. Infliximab is approved for several rheumatologic indications in adulthood including rheumatoid arthritis (RA), PsA, ankylosing spondylitis, and plaque psoriasis, but is not approved for any PiRD indication so far. In paediatrics, infliximab is still restricted for in-label use in paediatric chronic inflammatory bowel diseases. Furthermore, there are some differences between the FDA and EMA in bDMARDs and JAK approvals. For example, the FDA has approved rilonacept for the treatment of the Cryopyrin-associated periodic syndrome (CAPS) in adults and children aged 12 years and older, whereas EMA has not. Particularly relevant for the PiRD patients are the different age limitations for different bDMARDs, and varying age restriction for different PiRD diagnoses. No bDMARDs are approved in children younger than 2 years with the exception of anakinra, which is approved by the EMA for the age ≥ 8 months. The age limitations have changed over the last couple of years, and today the common age categories are ≥2, ≥4, ≥6, or ≥ 12 years. A detailed overview about paediatric bDMARDs approvals, indications and age limitations by the EMA and FDA in March 2020 compared with adult rheumatology is given in Table 6.

**Table 6:** Overview of bDMARDs and JAK inhibitors approved in adult and paediatric rheumatology (March 2020)

Drug class	Drug (brand name)	Adults		Children			
		Approved by FDA (date)	Approved by EMA (date)	Approved by FDA (date)	Current FDA age criteria	Approved by EMA (date)	Current EMA age criteria
**Anti-CD20 agent**	rituximab^a^ (MabThera, Rituxan)	RA (2006), WG/MPA (2011)	RA(2006), GPA/MPA (2013)	GPA/MPA (2019)	≥2 years	GPA/MPA (2020)	≥2 years
**CD80/86 inhibitor**	abatacept (Orencia)	RA (2005), PsA (2017)	RA (2007), PsA (2017)	PJIA (2008)	≥2 years (sc); ≥6 years (iv)	PJIA (2009)	≥2 years
**IL-1 inhibitor**	anakinra (Kineret)	RA (2001) CINCA/NOMID (2012)	RA (2002), CAPS (2013), AOSD (2018)	NOMID/CINCA (2012)	NA	CAPS (2013), SJIA (2018)	≥8 months
	canakinumab (Ilaris)	CAPS (2009), TRAPS/MKD/FMF (2016)	CAPS (2009), AOSD (2016), TRAPS/FMF/MKD (2016)	CAPS (2009), SJIA (2013), TRAPS/ FMF/MKD (2016)	≥4 years CAPS/TRAPS/MKD/ FMF; ≥2 years SJIA	CAPS (2009), SJIA (2013), TRAPS/FMF/ MKD (2016)	≥2 years
	rilonacept (Arcalyst)	CAPS (2008)	not approved	CAPS (2008)	≥12 years	not approved	
**IL-6 inhibitor**	tocilizumab^b^ (RoAcetemra/Actemra)	RA (2010)	RA (2008)	SJIA (2011), PJIA (2013)	≥2 years	SJIA (2011), PJIA (2013)	≥1 years SJIA; ≥2 years PJIA
	sarilumab (Kevzara)	RA (2017)	RA (2017)	not approved		not approved	
**IL-12/23 inhibitor**	ustekinumab^c^ (Stelara)	Plaque psoriasis (2009), PsA (2013)	Plaque psoriasis (2008), PsA (2014)	Plaque psoriasis (2017)	≥12 years	Plaque psoriasis (2015)	≥6 years
**IL-23 inhibitor**	guselkumab (Tremfya)	Plaque psoriasis (2017)	Plaque psoriasis (2017)	not approved		not approved	
	risankizumab (Skyrizi)	Plaque psoriasis (2019)	Plaque psoriasis (2019)	not approved		not approved	
	tildrakizumab (Ilumya/Ilumetri)	Plaque psoriasis (2018)	Plaque psoriasis (2018)	not approved		not approved	
**IL-17 inhibitor**	brodalumab (Siliq, Kyntheum)	Plaque psoriasis (2017)	Plaque psoriasis (2017)	not approved		not approved	
	ixekizumab (Taltz)	Plaque psoriasis (2016), PsA (2017), AS (2019)	Plaque psoriasis (2016), PsA (2017)	Plaque psoriasis (2020)	≥6 years	not approved	
	secukinumab (Cosentyx)	Plaque psoriasis (2015), AS (2016), PsA (2016)	Plaque psoriasis (2014), PsA (2015), AS (2015)	not approved		not approved	
**TNF inhibitor**	adalimumab^d^ (Humira)	RA (2002), PsA (2005), AS (2006), plaque psoriasis (2008), non-infectious intermediate, posterior and panuveitis (2016)	RA (2003), PsA (2005), AS (2006), plaque psoriasis (2007), non-radiographic axial spondyloarthritis (2012), non-infectious intermediate, posterior and panuveitis (2016)	PJIA (2008)	≥2 years	PJIA (2008), ERA (2014), plaque psoriasis (2015), non-infectious anterior uveitis (2017)	≥2 years PJIA; ≥2 years uveitis; ≥4 years plaque psoriasis; ≥6 years ERA
	certolizumab pegol^e^ (Cimzia)	RA (2009), PsA (2013), AS (2013), plaque psoriasis (2018), non-radiographic axial spondyloarthritis (2019)	RA (2009), PsA (2013), AS/non-radiographic axial spondyloarthritis (2013), plaque psoriasis (2018)	not approved		not approved	
	etanercept (Enbrel)	RA (1998), PsA (2002), AS (2003), plaque psoriasis (2004)	RA (2000), PsA (2002), AS (2004), plaque psoriasis (2004), non-radiographic axial spondyloarthritis (2014)	PJIA (1999), plaque psoriasis (2016)	≥2 years PJIA; ≥4 years plaque psoriasis	PJIA (2001), plaque psoriasis (2008), ERA/PsA (2012)	≥2 years PJIA; ≥6 years plaque psoriasis; ≥12 years ERA/PsA
	golimumab^f^ (Simponi)	RA/PsA/AS (2009)	RA/PsA/AS (2009), non-radiographic axial spondyloarthritis (2015)	not approved		PJIA (2016)	≥2 years
	infliximab^g^ (Remicade)	RA (1999), AS (2004), PsA (2005), plaque psoriasis (2006)	RA (2000), AS (2003), PsA (2004), plaque psoriasis (2005)	not approved		not approved	
**BAFF inhibitor**	belimumab (Benlysta)	SLE (2011)	SLE (2011)	SLE (2019)	≥5 years	SLE (2019)	≥5 years
**JAK inhibitor**	tofacitinib^8^ (Xeljanz)	RA (2012), PsA (2017)	RA (2017), PsA (2018)	not approved		not approved	
	baricitinib (Olumiant)	RA (2018)	RA (2016)	not approved		not approved	
	upadacitinib (Rinvoq)	RA (2019)	RA (2019)	not approved		not approved	

^a^Also approved to treat Non-Hodgkin’s Lymphoma, chronic lymphatic leukemia and pemphigus vulgaris; ^b^Also approved to treat giant cell arteritis, cytokine release syndrome (≥2 years); ^c^Also approved to treat ulcerative colitis (FDA only), Crohn’s disease; ^d^Also approved to treat ulcerative colitis, Crohn’s disease (≥6 years), hidradenitis suppurativa (age ≥ 12 years); ^e^Also approved to treat Crohn’s disease (FDA only); ^f^Also approved to treat ulcerative colitis; ^g^Also approved to treat Crohn’s disease (≥6 years), ulcerative colitis (≥6 years); ^h^Also approved to treat ulcerative colitis (FDA only)

*Abbreviations*: *AOSD* adult-onset still’s disease, *AS* ankylosing spondyloarthritis/spondylitis, *CAPS* cryopyrin-associated periodic syndrome, *CINCA* chronic infantile neurologic, cutaneous, and arthritis, *EMA* European Medicines Agency, *ERA* enthesitis-related juvenile idiopathic arthritis, *FDA* Food and Drug Administration, *FMF* familial mediterranean fever, *GPA* granulomatosis with polyangiitis, *IL* interleukin, *MKD* mevalonate kinase deficiency, *MPA* microscopic polyangiitis, *NA.* not applicable, *NOMID* neonatal-onset multisystem inflammatory disease, *PJIA* polyarticular juvenile idiopathic arthritis, *PsA* psoriatic arthritis/ psoriatic juvenile idiopathic arthritis, *RA* rheumatoid arthritis, *SJIA* systemic juvenile idiopathic arthritis, *SLE* systemic lupus erythematosus, TNF tumor necrosis factor receptor-associated periodic syndrome, *TRAPS* tumour necrosis factor receptor-associated periodic syndrome, *WG* Wegner’s Granulomatosis

## Discussion

This review indicates that reported data from RCTs characterizing efficacy, safety and/or PK, remains limited for several prescribed bDMARDs and JAK inhibitors in PiRD patients. As RCTs are robust research methods to determine cause-effect relationships between intervention and outcome, they are important to generate evidence in basic, translational and clinical research and can improve management of patients [112]. In the past, several clinical trials were conducted in PiRD with support of research networks in paediatric rheumatology, such as the Pediatric Rheumatology Collaborative Study Group (PRCSG) and the Paediatric Rheumatology International Trials Organisation (PRINTO) resulting in bDMARDs approval for some PiRD indications [19, 113]. This review indicates that TNF inhibitors are the most studied bDMARDs in PiRD patients, particularly in the JIA group. JIA is one of the most commonly diagnosed PiRDs with a prevalence of 16/100,000 to 150/100,000 [3]. In several JIA sub-groups, treatment with TNF inhibition is recommended, particularly when conventional disease modifying antirheumatic drugs (cDMARDs) cannot achieve the defined target [114, 115]. One of the first FDA-approved TNF inhibitors for polyarticular JIA treatment was etanercept in 1999, followed by adalimumab in 2008. This might explain why a majority of RCTs were performed for etanercept. Up to now, no JAK inhibitor is approved for PiRD patients. JAK inhibitors can be administered orally and therefore this treatment approach might be of particular interest in paediatric rheumatology, explaining why several RCTs are currently performed for JAK inhibitors. For several bDMARDs, a latency in drug approval for PiRD patients can be observed with a delay ranging between 1 year to 9 years. However, around 50% of reviewed therapeutic drugs are currently not approved for PiRD patients. Off-label and unlicensed drug use is frequent in paediatric patient populations [116, 117] and a considerable number of PiRD patients has to be treated with off-label bDMARDs or JAK inhibitors as no approved drugs are available for their age group, the PiRD indication or in general [20–23, 25]. Off-label use is often of great concern to the families of the affected children [17]. In addition, it seems that off-label and unlicensed drug use in children is associated with increased risk of medication errors and adverse events [118–120]. As infants and children with PiRD differ greatly from adult rheumatology patients the lack of paediatric PK data for bDMARDs and JAK inhibitors, can result in over- and under-dosing [42, 47, 121]. While under-dosing/low drug concentrations can result in drug-antibodies and drug insufficiency with uncontrolled chronic inflammation and disease burden, over-dosing can be associated with serious short- and long-term safety events [122–124]. There are data suggesting that based on the body weight, the clearance of several drugs is higher in paediatrics than in adults [39]. In PiRD patients, particularly in infants and younger children, there are data for bDMARDs and JAKs indicating a need for more frequent drug administration due to shorter half-life or the need for higher weight based drug dosages to achieve the defined therapy target [121, 125–127]. Moreover, it seems that subcutaneously administered bDMARDs are absorbed faster in young children [44]. As the therapy outcomes in PiRD patients is influenced by these age-dependent PK processes and the disease course, it is crucial to understand the developmental changes to optimize bDMARDs and JAK inhibitor dosing in paediatric rheumatology [39–42, 44, 46, 47]. As a consequence, the FDA Modernization Act stimulates the conduct of dedicated clinical studies to enhance understanding of PK, efficacy-safety balance, and optimal dosing of drugs in paediatric patients [128]. Nevertheless, concern has been raised that trial discontinuation, and nonpublication with associated risk of publication bias, seems to be common in paediatric patients [129, 130]. Slow recruitment rates in rare paediatric diseases can be challenging for paediatric trials, and poor recruitment seems to be one of the major risks for early termination or discontinuation of such studies [130]. These observations highlight the value of established research networks in paediatric rheumatology, such as PRCSG and PRINTO, in conducting clinical studies in PiRD patients as efficacy, safety and PK data obtained from PiRD patients to optimize treatment are warranted.

This review has several limitations. Despite a comprehensive search strategy and independent reviewer processes, there might be a risk of a reporting bias as unpublished RCTs were not included. Furthermore, this review does not include observational studies, single arm studies or RCTs including both children and adults. We cannot rule out that not all conducted RCTs in PiRDs were identified, despite a rigorous screening and review process. We have included RCTs with patients aged 20 years and younger, although this upper age limit of 20 years constituted the risk of having studies performed mainly in adolescents and young adults. To address this bias we have reported for each analysed RCT the age criteria and the median/mean age. As several included RCTs had an upper age criteria between 17 to 20 years, we would have missed otherwise these studies if we have limited the search to the age criteria 16 or 18 years.

## Conclusion

In summary, paediatric rheumatology patients differ from adult rheumatology patients in many aspects. As therapeutic drug response is influenced by age-dependent PK processes and disease course, it is important to consider developmental changes when prescribing bDMARDs or JAK inhibitors in PiRD patients. As such, it is critical to conduct international multicentre studies in PiRD patients to enroll a sufficiently high patient number in a reasonable period of time with the goal to appropriately investigate and characterize PK, efficacy and safety for bDMARDs and JAK inhibitors. More efficacy and safety data, ideally combined with PK data from PiRD patients will optimize bDMARDs and JAK inhibitor use in paediatric rheumatology.

## Supplementary Information

**Additional file 1: Supplementary data S1.** Search terms. **Supplementary data S2.** Review protocol. **Supplemetary data S3.** Outcome/Endpoint inclusion criteria for literature search.

## Data Availability

not applicable.

## Abbreviations

AID: Autoinflammatory diseases
cDMARDS: conventional disease modifying antirheumatic drugs
bDMARDS: Biologic disease modifying antirheumatic drugs
CTD: Connective tissue diseases
EMA: European Medicines Agency
ERA: Enthesitis-related juvenile idiopathic arthritis
FDA: Food and Drug Administration
ILAR: International League of Associations for Rheumatology
IL: Interleukin
JAK: Janus kinase
JIA: Juvenile idiopathic arthritis
KD: Kawasaki disease
NSAID: Non-steroidal anti-inflammatory drugs
OJIA: Oligoarticular juvenile idiopathic arthritis
PiRD: Pediatric inflammatory rheumatic diseases
PJIA: Polyarticular juvenile idiopathic arthritis
PK: Pharmacokinetics
PsA: Psoriatic juvenile idiopathic arthritis
RA: Rheumatoid arthritis
RF: Rheumatoid factor
RCT: Randomized controlled trials
SJIA: Systemic juvenile idiopathic arthritis
SLE: Systemic lupus erythematosus
TNF: Tumour necrosis factor
T2T: Treat to target

## References

[1] Teague M (2017). Pediatric rheumatologic diseases: a review for primary care NPs. Nurse Pract.

[2] Petty RE, Southwood TR, Manners P, Baum J, Glass DN, Goldenberg J (2004). International league of associations for rheumatology classification of juvenile idiopathic arthritis: second revision, Edmonton, 2001. J Rheumatol.

[3] Ravelli A, Martini A (2007). Juvenile idiopathic arthritis. Lancet..

[4] Ruperto N, Martini A (2004). International research networks in pediatric rheumatology: the PRINTO perspective. Curr Opin Rheumatol.

[5] Ruperto N, Ravelli A, Falcini F, Lepore L, Buoncompagni A, Gerloni V (1999). Responsiveness of outcome measures in juvenile chronic arthritis. Italian Pediatric Rheumatology Study Group. Rheumatology.

[6] Consolaro A, Giancane G, Schiappapietra B, Davi S, Calandra S, Lanni S (2016). Clinical outcome measures in juvenile idiopathic arthritis. Pediatr Rheumatol Online J..

[7] Consolaro A, Ruperto N, Bazso A, Pistorio A, Magni-Manzoni S, Filocamo G (2009). Development and validation of a composite disease activity score for juvenile idiopathic arthritis. Arthritis Rheum.

[8] Ravelli A, Consolaro A, Horneff G, Laxer RM, Lovell DJ, Wulffraat NM (2018). Treating juvenile idiopathic arthritis to target: recommendations of an international task force. Ann Rheum Dis.

[9] Hinze CH, Oommen PT, Dressler F, Urban A, Weller-Heinemann F, Speth F (2018). Development of practice and consensus-based strategies including a treat-to-target approach for the management of moderate and severe juvenile dermatomyositis in Germany and Austria. Pediatr Rheumatol Online J..

[10] Hansmann S, Lainka E, Horneff G, Holzinger D, Rieber N, Jansson AF (2020). Consensus protocols for the diagnosis and management of the hereditary autoinflammatory syndromes CAPS, TRAPS and MKD/HIDS: a German PRO-KIND initiative. Pediatr Rheumatol Online J.

[11] Smolen JS (2012). Treat-to-target: rationale and strategies. Clin Exp Rheumatol.

[12] McCoy SS, Stannard J, Kahlenberg JM (2016). Targeting the inflammasome in rheumatic diseases. Transl Res.

[13] Ruperto N, Martini A (2018). Current and future perspectives in the management of juvenile idiopathic arthritis. Lancet Child Adolesc Health.

[14] Maggi L, Mazzoni A, Cimaz R, Liotta F, Annunziato F, Cosmi L (2019). Th17 and Th1 lymphocytes in Oligoarticular juvenile idiopathic arthritis. Front Immunol.

[15] Jesus AA, Goldbach-Mansky R (2014). IL-1 blockade in autoinflammatory syndromes. Annu Rev Med.

[16] Vanoni F, Minoia F, Malattia C (2017). Biologics in juvenile idiopathic arthritis: a narrative review. Eur J Pediatr.

[17] Sterba Y, Ilowite N (2016). Biologics in pediatric rheumatology: quo Vadis?. Curr Rheumatol Rep.

[18] Garg S, Wynne K, Omoyinmi E, Eleftheriou D, Brogan P (2019). Efficacy and safety of anakinra for undifferentiated autoinflammatory diseases in children: a retrospective case review. Rheumatol Adv Pract.

[19] Brunner HI, Rider LG, Kingsbury DJ, Co D, Schneider R, Goldmuntz E (2018). Pediatric rheumatology collaborative study group - over four decades of pivotal clinical drug research in pediatric rheumatology. Pediatr Rheumatol Online J..

[20] Jung JY, Kim MY, Suh CH, Kim HA (2018). Off-label use of tocilizumab to treat non-juvenile idiopathic arthritis in pediatric rheumatic patients: a literature review. Pediatr Rheumatol Online J..

[21] Vitale A, Insalaco A, Sfriso P, Lopalco G, Emmi G, Cattalini M (2016). A snapshot on the on-label and off-label use of the Interleukin-1 inhibitors in Italy among rheumatologists and pediatric rheumatologists: a Nationwide multi-center retrospective observational study. Front Pharmacol.

[22] Sanchez GAM, Reinhardt A, Ramsey S, Wittkowski H, Hashkes PJ, Berkun Y (2018). JAK1/2 inhibition with baricitinib in the treatment of autoinflammatory interferonopathies. J Clin Invest.

[23] Gomez-Garcia F, Sanz-Cabanillas JL, Viguera-Guerra I, Isla-Tejera B, Nieto AV, Ruano J (2018). Scoping review on use of drugs targeting interleukin 1 pathway in DIRA and DITRA. Dermatol Ther (Heidelb).

[24] Woerner A, Belot A, Merlin E, Wouters C, Berthet G, Kondi A (2014). Prescribed but not approved: biologic agents used without approval in juvenile idiopathic arthritis in Switzerland, France and Belgium. Pediatr Rheumatol.

[25] Boyadzhiev M, Marinov L, Boyadzhiev V, Iotova V, Aksentijevich I, Hambleton S (2019). Disease course and treatment effects of a JAK inhibitor in a patient with CANDLE syndrome. Pediatr Rheumatol Online J..

[26] Picco P, Brisca G, Traverso F, Loy A, Gattorno M, Martini A (2009). Successful treatment of idiopathic recurrent pericarditis in children with interleukin-1beta receptor antagonist (anakinra): an unrecognized autoinflammatory disease?. Arthritis Rheum.

[27] Ozen S, Sonmez HE, Demir S (2018). Pediatric forms of vasculitis. Best Pract Res Clin Rheumatol.

[28] Lei C, Huang Y, Yuan S, Chen W, Liu H, Yang M, et al. Takayasu Arteritis With Coronary Artery Involvement: Differences Between Pediatric and Adult Patients. Can J Cardiol. 2019. 10.1016/j.cjca.2019.08.039 31924450

[29] Condie D, Grabell D, Jacobe H (2014). Comparison of outcomes in adults with pediatric-onset morphea and those with adult-onset morphea: a cross-sectional study from the morphea in adults and children cohort. Arthritis Rheumatol..

[30] Tarr T, Derfalvi B, Gyori N, Szanto A, Siminszky Z, Malik A (2015). Similarities and differences between pediatric and adult patients with systemic lupus erythematosus. Lupus..

[31] Fonseca R, Aguiar F, Rodrigues M, Brito I (2018). Clinical phenotype and outcome in lupus according to age: a comparison between juvenile and adult onset. Reumatol Clin.

[32] Panupattanapong S, Stwalley DL, White AJ, Olsen MA, French AR, Hartman ME (2018). Epidemiology and outcomes of Granulomatosis with Polyangiitis in pediatric and working-age adult populations in the United States: analysis of a large National Claims Database. Arthritis Rheumatol.

[33] Denby KJ, Clark DE, Markham LW (2017). Management of Kawasaki disease in adults. Heart..

[34] Piram M, Mahr A (2013). Epidemiology of immunoglobulin a vasculitis (Henoch-Schonlein): current state of knowledge. Curr Opin Rheumatol.

[35] Girschick H, Finetti M, Orlando F, Schalm S, Insalaco A, Ganser G (2018). The multifaceted presentation of chronic recurrent multifocal osteomyelitis: a series of 486 cases from the Eurofever international registry. Rheumatology..

[36] Rigante D, Vitale A, Natale MF, Lopalco G, Andreozzi L, Frediani B (2017). A comprehensive comparison between pediatric and adult patients with periodic fever, aphthous stomatitis, pharyngitis, and cervical adenopathy (PFAPA) syndrome. Clin Rheumatol.

[37] Constantin T, Foeldvari I, Anton J, de Boer J, Czitrom-Guillaume S, Edelsten C (2018). Consensus-based recommendations for the management of uveitis associated with juvenile idiopathic arthritis: the SHARE initiative. Ann Rheum Dis.

[38] McCrindle BW, Rowley AH, Newburger JW, Burns JC, Bolger AF, Gewitz M (2017). Diagnosis, treatment, and long-term Management of Kawasaki Disease: a scientific statement for health professionals from the American Heart Association. Circulation..

[39] Mahmood I (2016). Pharmacokinetic considerations in designing pediatric studies of proteins, antibodies, and plasma-derived products. Am J Ther.

[40] van den Anker J, Reed MD, Allegaert K, Kearns GL (2018). Developmental changes in pharmacokinetics and pharmacodynamics. J Clin Pharmacol.

[41] Kearns GL, Abdel-Rahman SM, Alander SW, Blowey DL, Leeder JS, Kauffman RE (2003). Developmental pharmacology--drug disposition, action, and therapy in infants and children. N Engl J Med.

[42] Samardzic J, Allegaert K, Bajcetic M (2015). Developmental pharmacology: a moving target. Int J Pharm.

[43] Nigrovic PA, Raychaudhuri S, Thompson SD (2018). Review: genetics and the classification of arthritis in adults and children. Arthritis Rheumatol..

[44] Malik P, Edginton A (2018). Pediatric physiology in relation to the pharmacokinetics of monoclonal antibodies. Expert Opin Drug Metab Toxicol.

[45] Moore P (1998). Children are not small adults. Lancet.

[46] Allegaert K (2018). Developmental Pharmacology - Special Issues During Childhood and Adolescence. Drug Res (Stuttg).

[47] Renton WD, Ramanan AV (2020). Better pharmacologic data the key to optimizing biological therapies in children. Rheumatology..

[48] Ruperto N, Lovell DJ, Quartier P, Paz E, Rubio-Perez N, Silva CA (2008). Abatacept in children with juvenile idiopathic arthritis: a randomised, double-blind, placebo-controlled withdrawal trial. Lancet..

[49] Ruperto N, Lovell DJ, Li T, Sztajnbok F, Goldenstein-Schainberg C, Scheinberg M (2010). Abatacept improves health-related quality of life, pain, sleep quality, and daily participation in subjects with juvenile idiopathic arthritis. Arthritis Care Res.

[50] Ruperto N, Lovell DJ, Quartier P, Paz E, Rubio-Perez N, Silva CA (2010). Long-term safety and efficacy of abatacept in children with juvenile idiopathic arthritis. Arthritis Rheum.

[51] Ruperto N, Lovell D, Bohnsack J, Breedt J, Fischbach M, Lutz T, et al. Subcutaneous or IntravenousAbatacept Monotherapy in Pediatric Patients with Polyarticular-Course JIA: Results from Two Phase III Trials [abstract]. Arthritis Rheumatol. 2019; 71 (suppl 10). https://acrabstracts.org/abstract/subcutaneous-or-intravenous-abatacept-monotherapy-in-pediatric-patients-with-polyarticular-course-jia-results-from-two-phase-iii-trials/. Accessed 23 Mar 2020.

[52] Ilowite N, Porras O, Reiff A, Rudge S, Punaro M, Martin A (2009). Anakinra in the treatment of polyarticular-course juvenile rheumatoid arthritis: safety and preliminary efficacy results of a randomized multicenter study. Clin Rheumatol.

[53] European Medicines Agency (2018). CHMP extension of indication variation assessment report (anakinra).

[54] Quartier P, Allantaz F, Cimaz R, Pillet P, Messiaen C, Bardin C (2011). A multicentre, randomised, double-blind, placebo-controlled trial with the interleukin-1 receptor antagonist anakinra in patients with systemic-onset juvenile idiopathic arthritis (ANAJIS trial). Ann Rheum Dis.

[55] Ruperto N, Brunner HI, Quartier P, Constantin T, Wulffraat N, Horneff G (2012). Two randomized trials of canakinumab in systemic juvenile idiopathic arthritis. N Engl J Med.

[56] Food US, Administration D (2016). Medical review and evaluation (canakinumab).

[57] European Medicines Agency (2014). Summary of product characteristics (canakinumab).

[58] ClinicalTrials.gov. Bethesda (MD): National Library of Medicine (US). 2000 Feb 29 - . Identifier NCT00534495, Safety and Effectiveness of Rilonacept for Treating Systemic Juvenile Idiopathic Arthritis in Children and Young Adults; 2007 Sept 26 [cited 2020 July 15]; [about 14 screens]. Available from: https://clinicaltrials.gov/ct2/show/results/NCT00534495

[59] Ilowite NT, Prather K, Lokhnygina Y, Schanberg LE, Elder M, Milojevic D (2014). Randomized, double blind, placebo-controlled trial of the efficacy and safety of rilonacept in the treatment of systemic juvenile idiopathic arthritis. Arthritis Rheumatol.

[60] Lovell DJ, Giannini EH, Reiff AO, Kimura Y, Li S, Hashkes PJ (2013). Long-term safety and efficacy of rilonacept in patients with systemic juvenile idiopathic arthritis. Arthritis Rheum.

[61] Brunner HI, Ruperto N, Zuber Z, Keane C, Harari O, Kenwright A (2015). Efficacy and safety of tocilizumab in patients with polyarticular-course juvenile idiopathic arthritis: results from a phase 3, randomised, double-blind withdrawal trial. Ann Rheum Dis.

[62] ClinicalTrials.gov. Bethesda (MD): National Library of Medicine (US). 2000 Feb 29 - . Identifier NCT00988221, A Study of Tocilizumab in Patients With Active Polyarticular Juvenile Idiopathic Arthritis; 2009 Oct 2 [cited 2020 July 15]; [about 50 screens]. Available from: https://clinicaltrials.gov/ct2/show/results/NCT00988221

[63] Brunner H, Chen C, Martini A, Espada G, Joos R, Akikusa J, et al. Disability and Health-Related Quality of Life Outcomes in Patients with Systemic or Polyarticular Juvenile Idiopathic Arthritis Treated with Tocilizumab in Randomized Controlled Phase 3 Trials [abstract]. Arthritis Rheumatol. 2019; 71 (suppl 10). https://acrabstracts.org/abstract/disability-and-health-related-quality-of-life-outcomes-in-patients-with-systemic-or-polyarticular-juvenile-idiopathic-arthritis-treated-with-tocilizumab-in-randomized-controlled-phase-3-trials/. Accessed 23 Mar 2020.

[64] US Food and Drug Administration. Medical review and evaluation (tocilizumab), 2013. https://www.accessdata.fda.gov/drugsatfda_docs/nda/2013/125276Orig1s064.pdf. (23 March 2020, date last accessed)

[65] European Medicines Agency. Assessment report (tocilizumab), 2013. https://www.ema.europa.eu/en/documents/variation-report/roactemra-h-c-955-ii-0026-epar-assessment-report-variation_en.pdf (23 March 2020, date last accessed).

[66] De Benedetti F, Brunner HI, Ruperto N, Kenwright A, Wright S, Calvo I (2012). Randomized trial of tocilizumab in systemic juvenile idiopathic arthritis. N Engl J Med.

[67] ClinicalTrials.gov. Bethesda (MD): National Library of Medicine (US). 2000 Feb 29 - . Identifier NCT00642460, A Study of RoActemra/Actemra (Tocilizumab) in Patients With Active Systemic Juvenile Idiopathic Arthritis (JIA); 2008 Mar 25 [cited 2020 July 15]; [about 64 screens]. Available from: https://clinicaltrials.gov/ct2/show/results/NCT00642460

[68] Yokota S, Imagawa T, Mori M, Miyamae T, Aihara Y, Takei S (2008). Efficacy and safety of tocilizumab in patients with systemic-onset juvenile idiopathic arthritis: a randomised, double-blind, placebo-controlled, withdrawal phase III trial. Lancet..

[69] Burgos-Vargas R, Tse SM, Horneff G, Pangan AL, Kalabic J, Goss S (2015). A randomized, double blind, placebo-controlled multicenter study of Adalimumab in pediatric patients with Enthesitis-related arthritis. Arthritis Care Res..

[70] European Medicines Agency (2016). Assessment report for paediatric studies submitted according to Article 46 of the Regulation (EC) No 1901/2006 (adalimumab).

[71] European Medicines Agency (2014). CHMP extension of indication variation assessment report (adalimumab).

[72] Horneff G, Fitter S, Foeldvari I, Minden K, Kuemmerle-Deschner J, Tzaribacev N (2012). Double blind, placebo-controlled randomized trial with adalimumab for treatment of juvenile onset ankylosing spondylitis (JoAS): significant short term improvement. Arthritis Res Ther.

[73] Lovell DJ, Ruperto N, Goodman S, Reiff A, Jung L, Jarosova K (2008). Adalimumab with or without methotrexate in juvenile rheumatoid arthritis. N Engl J Med.

[74] European Medicines Agency (2011). Assessment report (adalimumab).

[75] Ramanan AV, Dick AD, Jones AP, McKay A, Williamson PR, Compeyrot-Lacassagne S (2017). Adalimumab plus methotrexate for uveitis in juvenile idiopathic arthritis. N Engl J Med.

[76] European Medicines Agency (2017). Assessment report (adalimumab).

[77] Quartier P, Baptiste A, Despert V, Allain-Launay E, Kone-Paut I, Belot A (2018). ADJUVITE: a double blind, randomised, placebo-controlled trial of adalimumab in early onset, chronic, juvenile idiopathic arthritis-associated anterior uveitis. Ann Rheum Dis.

[78] Horneff G, Foeldvari I, Minden K, Trauzeddel R, Kummerle-Deschner JB, Tenbrock K (2015). Efficacy and safety of etanercept in patients with the enthesitis-related arthritis category of juvenile idiopathic arthritis: results from a phase III randomized, double-blind study. Arthritis Rheumatol.

[79] Lovell DJ, Giannini EH, Reiff A, Cawkwell GD, Silverman ED, Nocton JJ (2000). Etanercept in children with polyarticular juvenile rheumatoid arthritis. Pediatric rheumatology collaborative study group. N Engl J Med.

[80] US Food and Drug Administration (1999). Medical review and evaluation (etanercept).

[81] ClinicalTrials.gov. Bethesda (MD): National Library of Medicine (US). 2000 Feb 29 - . Identifier NCT03780959, Safety and Efficacy of Etanercept (Recombinant Human Tumor Necrosis Factor Receptor Fusion Protein [TNFR:Fc]) in Children With Juvenile Rheumatoid Arthritis (JRA); 2018 Dec 19 [cited 2020 July 15]; [about 12 screens]. Available from: https://clinicaltrials.gov/ct2/show/results/NCT03780959

[82] ClinicalTrials.gov. Bethesda (MD): National Library of Medicine (US). 2000 Feb 29 - . Identifier NCT03781375, Etanercept Plus Methotrexate Versus Methotrexate Alone in Children With Polyarticular Course Juvenile Rheumatoid Arthritis; 2018 Dec 19 [cited 2020 July 15]; [about 13 screens]. Available from: https://clinicaltrials.gov/ct2/show/results/NCT03781375

[83] Wallace CA, Giannini EH, Spalding SJ, Hashkes PJ, O'Neil KM, Zeft AS (2012). Trial of early aggressive therapy in polyarticular juvenile idiopathic arthritis. Arthritis Rheum.

[84] Wallace CA, Giannini EH, Spalding SJ, Hashkes PJ, O'Neil KM, Zeft AS (2014). Clinically inactive disease in a cohort of children with new-onset polyarticular juvenile idiopathic arthritis treated with early aggressive therapy: time to achievement, total duration, and predictors. J Rheumatol.

[85] ClinicalTrials.gov. Bethesda (MD): National Library of Medicine (US). 2000 Feb 29 - . Identifier NCT00078806, Safety and Efficacy Study of Etanercept (Enbrel®) In Children With Systemic Onset Juvenile Rheumatoid Arthritis; 2004 Mar 9 [cited 2020 July 15]; [about 24 screens]. Available from: https://clinicaltrials.gov/ct2/show/results/NCT00078806

[86] Hissink Muller PC, Brinkman DM, Schonenberg D, Koopman-Keemink Y, Brederije IC, Bekkering WP (2017). A comparison of three treatment strategies in recent onset non-systemic juvenile idiopathic arthritis: initial 3-months results of the BeSt for Kids-study. Pediatr Rheumatol Online J..

[87] Hissink Muller P, Brinkman DMC, Schonenberg-Meinema D, van den Bosch WB, Koopman-Keemink Y, Brederije ICJ (2019). Treat to target (drug-free) inactive disease in DMARD-naive juvenile idiopathic arthritis: 24-month clinical outcomes of a three-armed randomised trial. Ann Rheum Dis.

[88] Brunner HI, Ruperto N, Tzaribachev N, Horneff G, Chasnyk VG, Panaviene V (2018). Subcutaneous golimumab for children with active polyarticular-course juvenile idiopathic arthritis: results of a multicentre, double-blind, randomised-withdrawal trial. Ann Rheum Dis.

[89] European Medicines Agency (2016). Assessment report (golimumab).

[90] Ruperto N, Lovell DJ, Cuttica R, Wilkinson N, Woo P, Espada G (2007). A randomized, placebo controlled trial of infliximab plus methotrexate for the treatment of polyarticular-course juvenile rheumatoid arthritis. Arthritis Rheum.

[91] Tynjala P, Vahasalo P, Tarkiainen M, Kroger L, Aalto K, Malin M (2011). Aggressive combination drug therapy in very early polyarticular juvenile idiopathic arthritis (ACUTE-JIA): a multicentre randomised open-label clinical trial. Ann Rheum Dis.

[92] Brunner H, Synoverska O, Ting T, Abud Mendoza C, Spindler A, Vyzhga Y, et al. Tofacitinib for the Treatment of Polyarticular Course Juvenile Idiopathic Arthritis: Results of a Phase 3 Randomized, Double-blind, Placebo-controlled Withdrawal Study [abstract]. Arthritis Rheumatol. 2019;71(suppl 10) https://acrabstracts.org/abstract/tofacitinib-for-the-treatment-of-polyarticular-course-juvenile-idiopathic-arthritis-results-of-a-phase-3-randomized-double-blind-placebo-controlled-withdrawal-study/. Accessed 23 Mar 2020.

[93] Landells I, Marano C, Hsu MC, Li S, Zhu Y, Eichenfield LF (2015). Ustekinumab in adolescent patients age 12 to 17 years with moderate-to-severe plaque psoriasis: results of the randomized phase 3 CADMUS study. J Am Acad Dermatol.

[94] ClinicalTrials.gov. Bethesda (MD): National Library of Medicine (US). 2000 Feb 29 - . Identifier NCT03073200, Study of Ixekizumab (LY2439821) in Children 6 to Less Than 18 Years With Moderate-to-Severe Plaque Psoriasis (Ixora-peds); 2017 Mar 08 [cited 2020 July 15]; [about 32 screens]. Available from: https://clinicaltrials.gov/ct2/show/results/NCT03073200

[95] Papp K, Thaci D, Marcoux D, Weibel L, Philipp S, Ghislain PD (2017). Efficacy and safety of adalimumab every other week versus methotrexate once weekly in children and adolescents with severe chronic plaque psoriasis: a randomised, double-blind, phase 3 trial. Lancet..

[96] European Medicines Agency (2015). Assessment report for paediatric studies submitted according to Article 46 of the Regulation (EC) No 1901/2006 (adalimumab).

[97] European Medicines Agency (2015). Extension of indication variation assessment report (adalimumab).

[98] ClinicalTrials.gov. Bethesda (MD): National Library of Medicine (US). 2000 Feb 29 - . Identifier NCT01251614, A Double Blind Study in Pediatric Subjects With Chronic Plaque Psoriasis, Studying Adalimumab vs. Methotrexate); 2010 Dec 2 [cited 2020 July 15]; [about 48 screens]. Available from: https://clinicaltrials.gov/ct2/show/results/NCT01251614

[99] Portman MA, Dahdah NS, Slee A, Olson AK, Choueiter NF, Soriano BD, et al. Etanercept With IVIg for Acute Kawasaki Disease: A Randomized Controlled Trial. Pediatrics. 2019;143(6). 10.1542/peds.2018-3675 PMC6564061 31048415

[100] Paller AS, Siegfried EC, Langley RG, Gottlieb AB, Pariser D, Landells I (2008). Etanercept treatment for children and adolescents with plaque psoriasis. N Engl J Med.

[101] Langley RG, Paller AS, Hebert AA, Creamer K, Weng HH, Jahreis A (2011). Patient-reported outcomes in pediatric patients with psoriasis undergoing etanercept treatment: 12-week results from a phase III randomized controlled trial. J Am Acad Dermatol.

[102] Siegfried EC, Eichenfield LF, Paller AS, Pariser D, Creamer K, Kricorian G (2010). Intermittent etanercept therapy in pediatric patients with psoriasis. J Am Acad Dermatol.

[103] Landells I, Paller AS, Pariser D, Kricorian G, Foehl J, Molta C (2010). Efficacy and safety of etanercept in children and adolescents aged > or = 8 years with severe plaque psoriasis. Eur J Dermatol.

[104] European Medicines Agency (2008). Assessment report (etanercept).

[105] Han CL, Zhao SL (2018). Intravenous immunoglobulin gamma (IVIG) versus IVIG plus infliximab in young children with Kawasaki disease. Med Sci Monit.

[106] Mori M, Hara T, Kikuchi M, Shimizu H, Miyamoto T, Iwashima S (2018). Infliximab versus intravenous immunoglobulin for refractory Kawasaki disease: a phase 3, randomized, open-label, active-controlled, parallel-group, multicenter trial. Sci Rep.

[107] Tremoulet AH, Jain S, Jaggi P, Jimenez-Fernandez S, Pancheri JM, Sun X (2014). Infliximab for intensification of primary therapy for Kawasaki disease: a phase 3 randomised, double-blind, placebo-controlled trial. Lancet..

[108] Jaggi P, Wang W, Dvorchik I, Printz B, Berry E, Kovalchin JP (2015). Patterns of fever in children after primary treatment for Kawasaki disease. Pediatr Infect Dis J.

[109] ClinicalTrials.gov. Bethesda (MD): National Library of Medicine (US). 2000 Feb 29 - . Identifier NCT00589628, Multi Center Prospective Registry of Infliximab Use for Childhood Uveitis; 2008 Jan 10 [cited 2020 July 15]; [about 7 screens]. Available from: https://clinicaltrials.gov/ct2/show/results/NCT00589628

[110] Nino A, Bass D, Eriksson G, Hammer A, Ji B, Quasny H, Roth D. Efficacy and Safety of Intravenous Belimumab in Children with Systemic Lupus Erythematosus: An Across-Trial Comparison with the Adult Belimumab Studies [abstract]. Arthritis Rheumatol. 2019; 71 (suppl 10). https://acrabstracts.org/abstract/efficacy-and-safety-of-intravenous-belimumab-in-children-with-systemic-lupus-erythematosus-an-across-trial-comparison-with-the-adult-belimumab-studies/. Accessed 23 Mar 2020.

[111] European Medicines Agency (2019). Assessment report (belimumab).

[112] Bhide A, Shah PS, Acharya G (2018). A simplified guide to randomized controlled trials. Acta Obstet Gynecol Scand.

[113] Ruperto N, Martini A (2011). Networking in paediatrics: the example of the Paediatric rheumatology international trials organisation (PRINTO). Arch Dis Child.

[114] Beukelman T, Patkar NM, Saag KG, Tolleson-Rinehart S, Cron RQ, DeWitt EM (2011). 2011 American College of Rheumatology recommendations for the treatment of juvenile idiopathic arthritis: initiation and safety monitoring of therapeutic agents for the treatment of arthritis and systemic features. Arthritis Care Res..

[115] Angeles-Han ST, Ringold S, Beukelman T, Lovell D, Cuello CA, Becker ML (2019). 2019 American College of Rheumatology/Arthritis Foundation guideline for the screening, monitoring, and treatment of juvenile idiopathic arthritis-associated uveitis. Arthritis Care Res..

[116] Moulis F, Durrieu G, Lapeyre-Mestre M (2018). Off-label and unlicensed drug use in children population. Therapie..

[117] Gore R, Chugh PK, Tripathi CD, Lhamo Y, Gautam S (2017). Pediatric off-label and unlicensed drug use and its implications. Curr Clin Pharmacol.

[118] Conroy S (2011). Association between licence status and medication errors. Arch Dis Child.

[119] Wimmer S, Neubert A, Rascher W (2015). The safety of drug therapy in children. Dtsch Arztebl Int.

[120] Bellis JR, Kirkham JJ, Thiesen S, Conroy EJ, Bracken LE, Mannix HL (2013). Adverse drug reactions and off-label and unlicensed medicines in children: a nested case-control study of inpatients in a pediatric hospital. BMC Med.

[121] Kim H, Brooks KM, Tang CC, Wakim P, Blake M, Brooks SR (2018). Pharmacokinetics, pharmacodynamics, and proposed dosing of the Oral JAK1 and JAK2 inhibitor Baricitinib in pediatric and young adult CANDLE and SAVI patients. Clin Pharmacol Ther.

[122] Bartelds GM, Krieckaert CL, Nurmohamed MT, van Schouwenburg PA, Lems WF, Twisk JW (2011). Development of antidrug antibodies against adalimumab and association with disease activity and treatment failure during long-term follow-up. Jama..

[123] Skrabl-Baumgartner A, Seidel G, Langner-Wegscheider B, Schlagenhauf A, Jahnel J (2019). Drug monitoring in long-term treatment with adalimumab for juvenile idiopathic arthritis-associated uveitis. Arch Dis Child.

[124] Nestorov I (2005). Clinical pharmacokinetics of tumor necrosis factor antagonists. J Rheumatol Suppl.

[125] Neven B, Marvillet I, Terrada C, Ferster A, Boddaert N, Couloignier V (2010). Long-term efficacy of the interleukin-1 receptor antagonist anakinra in ten patients with neonatal-onset multisystem inflammatory disease/chronic infantile neurologic, cutaneous, articular syndrome. Arthritis Rheum.

[126] Kuemmerle-Deschner JB, Hachulla E, Cartwright R, Hawkins PN, Tran TA, Bader-Meunier B (2011). Two-year results from an open-label, multicentre, phase III study evaluating the safety and efficacy of canakinumab in patients with cryopyrin-associated periodic syndrome across different severity phenotypes. Ann Rheum Dis.

[127] Kuemmerle-Deschner JB, Hofer F, Endres T, Kortus-Goetze B, Blank N, Weissbarth-Riedel E (2016). Real-life effectiveness of canakinumab in cryopyrin-associated periodic syndrome. Rheumatology..

[128] Roberts R, Rodriguez W, Murphy D, Crescenzi T (2003). Pediatric drug labeling: improving the safety and efficacy of pediatric therapies. Jama..

[129] Shamliyan T, Kane RL (2012). Clinical research involving children: registration, completeness, and publication. Pediatrics..

[130] Pica N, Bourgeois F. Discontinuation and Nonpublication of Randomized Clinical Trials Conducted in Children. Pediatrics. 2016;138(3). 10.1542/peds.2016-0223 PMC5005019 27492817

