# Correction to: Biologic disease modifying antirheumatic drugs and Janus kinase inhibitors in paediatric rheumatology – what we know and what we do not know from randomized controlled trials

**DOI:** 10.1186/s12969-021-00593-3

**Published:** 2021-08-16

**Authors:** Tatjana Welzel, Carolyn Winskill, Nancy Zhang, Andreas Woerner, Marc Pfister

**Affiliations:** 1 Paediatric Pharmacology and Pharmacometrics, University Children’s Hospital Basel (UKBB), University of Basel, Spitalstrasse 33, CH 4031 Basel, Switzerland; 2 Paediatric Rheumatology, University Children’s Hospital Basel (UKBB), University of Basel, Basel, Switzerland; 3 Integrated Drug Development, Certara LP, Princeton, NJ USA

**Correction to: Pediatr Rheumatol 19, 46 (2021)**

**https://doi.org/10.1186/s12969-021-00514-4**

Following publication of the original article [1], the authors identified some errors in the text of the article. Correct text parts should be as follows:

1. Results section in Abstract should read as follows: All 35 RCTs reported efficacy while 34/35 provided safety outcomes and 16/35 provided pharmacokinetic data.
2. Table 4 should have IL-1 inhibitor canakinumab 4mg/kg single dose
3. Results section text should be as follows: In march 2020, the FDA has approved all 23 reviewed drugs, including bDMARDs and JAK inhibitors for adult rheumatology, whereas the EMA has approved 22 (Table 6).

## References

[1] Welzel (2021). Biologic disease modifying antirheumatic drugs and Janus kinase inhibitors in paediatric rheumatology – what we know and what we do not know from randomized controlled trials. Pediatr Rheumatol.

