# Supplementary material for: Biologic disease modifying antirheumatic drugs and Janus kinase inhibitors in paediatric rheumatology – what we know and what we do not know from randomized controlled trials
Source: Pediatr Rheumatol Online J. 2021 Mar 25;19:46. doi: 10.1186/s12969-021-00514-4 (PMC7995584; doi:10.1186/s12969-021-00514-4)
Supplement: Supplementary file 1 — Additional file 1: Supplementary data S1. Search terms. Supplementary data S2. Review protocol. Supplemetary data S3. Outcome/Endpoint inclusion criteria for literature search. [file 12969_2021_514_MOESM1_ESM.docx]

**Supplementary material**

Supplementary data S1: Search terms

1. PubMed search terms

(pediatric OR children OR infants OR juvenile OR neonatal OR teen OR adolescent) AND (abatacept OR adalimumab OR anakinra OR baricitinib OR belimumab OR brodalumab OR canakinumab OR certolizumab OR etanercept OR golimumab OR guselkumab OR infliximab OR ixekizumab OR risankizumab OR rilanocept OR rituximab OR sarilumab OR secukinumab OR tildrakizumab OR tocilizumab OR tofacitinib OR upadacitinib OR ustekinumab OR Janus kinase OR JAK inhibitor) AND randomized controlled trial [publication type] AND English

1. Clinicaltrials.gov search terms

For each drug listed in the inclusion criteria, the following search filter was applied: Interventional Studies (Clinical Trials) AND Child (birth-17) AND Studies with Results. To find recruiting studies, the “Studies with Results” filter was not included in the search terms.

Supplementary data S2: Review protocol

1. Background

This review was performed to analyze what we know from randomized control trails (RCTs) in paediatrics with inflammatory rheumatic diseases (PiRD) patients treated with defined biologic disease modifying antirheumatic drugs (bDMARDs) and Janus kinase (JAK) inhibitors. The literature search was done systematically, based on relevant identification, screening, and assessment steps described in the Cochrane Handbook for Systematic Reviews of Interventions (1) and reporting items in the PRISMA statement (2).

1. Objective

The primary objective is to provide an independent depository of performed RCTs in PiRD patients treated with bDMARDs and JAK inhibitors.

1. Methods
   1. Eligibility

Titles and abstracts are screened for eligibility according to the pre-defined eligibility and exclusion criteria:

1. Eligibility criteria
   - Patients aged 20 years and younger
   - Treatment with predefined bDMARDs/JAK inhibitors
     - Anti-CD20 agents: rituximab
     - CD80/86 inhibitors: abatacept
     - IL-1 inhibitors: anakinra, canakinumab, rilonacept
     - IL-6 inhibitors: tocilizumab, sarilumab
     - IL-12/23 inhibitors: ustekinumab
     - IL-23 inhibitors: guselkumab, risankizumab, tildrakizumab
     - IL-17 inhibitors: secukinumab, ixekizumab, brodalumab
     - Tumour necrosis factor (TNF) inhibitors: adalimumab, etanercept, golimumab, infliximab, certolizumab pegol
     - BAFF inhibitors: belimumab
     - JAK inhibitors: baricitinib, tofacitinib, upadacitinib
   - Sample size ≥ five patients
   - Confirmed PiRD diagnosis (Main manuscript: table 1).
   - At least one relevant primary or secondary efficacy endpoint/outcome (Supplementary data S3).
   - English language
   - Published after 1990
2. Exclusion criteria
   - Indication not relevant
   - Population not relevant
   - Study design not relevant (not a RCT)
   - Treatment not relevant
   - Endpoint/outcome not relevant
   - Duplicate of prior published results without any additional information

3.2 Search and retrieval

The primary sources of information for this database were PubMed, the US National Institutes of Health Ongoing Trials Register ClinicalTrials.gov ([www.clinicaltrials.gov](http://www.clinicaltrials.gov)), and the EU Clinical Trials Register ([www.clinicaltrialsregister.eu](http://www.clinicaltrialsregister.eu)) augmented by searches on conference abstracts/posters/presentations (EULAR, PRES, ISSAID), as well as regulatory reviews from FDA/EMA websites (www.fda.gov, www.ema.europa.eu).

3.3 Identification

Identification of eligible study reports were based on search terms mentioned at the supplementary material data S1. Web searches for company trial registries were conducted for additional data for drugs under development. Finally, references list of published studies were reviewed to identify any additional references. The search results were exported and managed in a spreadsheet and electronic copies (PDFs) of study reports are retrieved via internet sources or local libraries.

3.4 Screening and eligibility assessment

Initial screening, based on retrieved abstracts, as well as the eligibility assessment based on full-text publications were performed by two scientists. One scientist was responsible for the execution and documentation and the other provided support as the therapeutic area expert.

3.5 Data extraction

Data extraction was done by scientists using an electronic data extraction spreadsheet developed in MS Excel. Consensus sessions were held regularly to resolve data extraction issues.

3.6 Final inclusion

Final selection of the study reports was performed after the scientists involved in the project reached a consensus.

***References***

1. Higgins JPT, Green S (editors). Cochrane Handbook for Systematic Reviews of Interventions Version 5.0.2 [updated September 2009]. The Cochrane Collaboration, 2009. Available from www.cochrane-handbook.org.
2. Moher D, Liberati A, Tetzlaff J, Altman DG, The PRISMA Group (2009). Preferred Reporting Items for Systematic Reviews and Meta-Analyses: The PRISMA Statement. PLoS Med 6(6): e1000097. doi:10.1371/journal.pmed1000097

Supplementary data S3

Outcome/Endpoint inclusion criteria for literature search

| Indication | Population | Outcome/Endpoints |
| --- | --- | --- |
| all paediatric inflammatory rheumatic diseases (PiRD) |  | Paediatric Quality of Life Inventory (PedsQoL)  C-reactive protein  Blood sedimentation rate  Kidney parameters (Proteinuria, Creatinine, eGFR)  S 100 proteins (S100A12, S100A9, S100A8)  Blood count  Hepathopathy/Hepatosplenomegaly  Organ damage (lung, esophagous, kidney)  Allergic reactions  Kidney injury  Infections  Cancer  Anti-drug-antibodies |
| Juvenile idiopathic arthritis (JIA) | Polyarticular rheumatoid factor positive/  negative JIA (PJIA)  Persistent or extended oligoarticular JIA (OJIA)  Enthesitis-related juvenile arthritis and juvenile ankylosing spondylitis, including sacroiliitis (ERA)  Psoriatic juvenile idiopathic arthritis; ( PsA)  Systemic JIA (SJIA) | ACR Pedi 30%/50%/70%/90% response criteria (ACR Pedi 30/50/70/90)  Assessment of Spondylarthritis International Society score (ASAS)Childhood Health Assessment Questionnaire (CHAQ)  Juvenile Arthritis Multidimensional Assessment Report (JAMAR)  Juvenile Arthritis Disease Activity Score (JADAS 10/27/71)  Juvenile Arthritis Damage Index (JADI)  Juvenile Arthritis Functional Assessment Report (JAFAR)  Radiographic Progression of the Disease (Genant or Modified Sharp Scores)  Morning stiffness  Physician Global assessment (PGA)  Active joints |
| Uveitis | JIA-uveitis  non-infectious uveitis | Visual loss  Degree of Tyndall  Degree of Uveitis  Improvement of Laser flare photometry (LFP) |
| Autoinflammatory Diseases | Familial Mediterranean Fever (FMF)  TNF receptor-1 associated periodic syndrome (TRAPS)  Cryopyrin-associated periodic syndromes (CAPS)  Mevalonate Kinase Deficiency (MKD) /Hyperimmunoglobulin D syndrome (HIDS)  Unclassified periodic fever syndromes  Chronic recurrent multifocal osteomyelitis (CRMO)  Majeed syndrome  Deficiency of the interleukin-1 receptor antagonist (DIRA)  A20 haploinsufficiency (HA20)  Sideroblastic anemia with B cell immunodeficiency, periodic fevers and developmental delay syndrome (SIFD)  Pyogenic arthritis, pyoderma gangraenosum and acne (PAPA)  Deficiency of the interleukin-36 receptor antagonist (DITRA)  Palmar plantar pustulosis (PPP)  Pyoderma gangraenosum  Chronic atypical neutrophilic dermatitis with lipodystrophy and elevated temperature (CANDLE)  Stimulator of interferon genes (STING)-associated vasculopathy with onset in infancy (SAVI) | Autoinflammatory Disease Activity Index (AIDAI)  Autoinflammatory Disease Damage Index (ADDI)  Serum Amyloid A (SAA)  Interleukin-6 (IL-6)  Interleukin-1 (IL-1)  Amyloidosis  Hearing loss  Physician Global assessment (PGA) |
| Vasculitis | Takayasu arteritis  Leucocytoclastic vasculitis  Granulomatosis with polyangiitis (GPA)  Wegener’s Granulomatosis  Polyarteritis nodosa  Microscopic polyangiitis (MPA)  Eosinophilic granulomatosis with polyangiitis  Kawasaki Disease (KD)  Behcet disease | Paediatric vasculitis activity score (PVAS) |
| Connective Tissue Diseases (CTD) | Systemic Lupus Erythematosus (SLE) | Systemic Lupus International Collaborating Clinics (SLICC)  British Isles Lupus Assessment Group (BILAG) Index 2004  Cytopenia  Systemic Lupus Erythematosus Response Index 4 (SRI4) |
|  | Juvenile Dermatomyositis (JDM) | Childhood Myositis Assessment Score (CMAS)  Creatininkinase  Aldolase  Myositis |
|  | Paediatric Sarcoidosis  Systemic Scleroderma  Localized Scleroderma  Sjögren Syndrome  Mixed connective tissue diseases (MCTD) |  |
| Macrophage activation syndrome |  |  |
| Psoriasis |  | PASI (Psoriasis Area and Severity Index) |
